# Current State of Resistance to Antibiotics of Last-Resort in South Africa: A Review from a Public Health Perspective

**DOI:** 10.3389/fpubh.2016.00209

**Published:** 2016-09-30

**Authors:** John Osei Sekyere

**Affiliations:** ^1^Department of Pharmaceutics, Division of Microbiology, Kwame Nkrumah University of Science and Technology, Kumasi, Ghana

**Keywords:** carbapenem, tigecycline, colistin, carbapenemase, *mcr-1*, South Africa, antibiotic resistance

## Abstract

A review of the literature was undertaken to delineate the current level and mechanisms of resistance to carbapenems, colistin, and tigecycline in South Africa. Thirty-two English publications and 32 National Institute of Communicable Diseases communiqués identified between early January 2000 and 20 May, 2016 showed substantial reports of NDM (*n* = 860), OXA-48 (*n* = 584), VIM (*n* = 131), and IMP (*n* = 45) carbapenemases within this period, mainly in *Klebsiella pneumoniae* (*n* = 1138), *Acinetobacter baumannii* (*n* = 332), *Enterobacter cloacae* (*n* = 201), and *Serratia marcescens* (*n* = 108). Colistin and tigecycline resistance was prevalent among *K. pneumoniae, A. baumannii, S. marcescens*, and *E. cloacae*. The first *mcr-1* colistin resistance gene to be detected in South Africa was reported in *Escherichia coli* from livestock as well as from hospitalized and outpatients. There are increasing reports of NDM and OXA-48 carbapenemases among Enterobacteriaceae and *A. baumannii* in South Africa. *Mcr-1* is now present in South African patients and livestock. Resistance to carbapenems, colistin, and tigecycline restricts infection management options for clinicians.

## Bullet Points/Highlights

- Resistance to last-resort antibiotics, such as carbapenems, tigecycline, and colistin, is increasing among Gram-negative bacteria in South Africa, restricting infection management options for clinicians and posing a threat to food safety.
- NDM-1 and OXA-48-like carbapenemases are the most common carbapenemases found among Gram-negative bacteria in South Africa and have been implicated in clinical outbreaks.
- The mcr-1 colistin resistance gene has been reported among *Escherichia coli* in both clinical and poultry samples in two provinces in South Africa. This is a threat to all Africa and Europe as many patients from several countries in these continents seek health care in South Africa.

## Introduction

The inexorable adaptations of bacteria to the therapeutic effects of antibiotics, with its grave implications, are dawning upon the world. This is witnessed in the increasing awareness being created by the WHO and EU about antibiotic resistance, specifically through the formers’ global/regional antibiotic surveillance reports (1, 2). Moreover, antibiotic resistance awareness days and weeks have been instituted by both organizations (3), while efforts to engage policy makers and governments (4), clinicians, and patients in antibiotic stewardship have increased concomitantly (1, 2). Until recently, the effect of antibiotic use in livestock production on clinical medicine was a subject of debate (5). However, the detection of *mcr-1* colistin resistance gene in swine, pork, imported chicken (in Denmark), and hospitalized patients has helped settle the argument that antibiotics use in veterinary medicine, specifically as growth promoters, can be a source of resistance genes in human pathogenic bacteria (6, 7). Subsequently, the concept of “One Health” that triangulates clinical, environmental, and veterinary antibiotic resistance surveillance and molecular epidemiological studies as a means to containing resistance is gaining grounds (8).

A recent study in the UK detected large amounts of resistance genes to clinically useful antibiotics, such as sulfonamides, trimethoprim, and tetracyclines, in rivers that were fed with runoff effluents from farms on which antibiotics were used, as well as from sewage processing plants (9). In India and Bangladesh, substantial amounts of broad-spectrum antibiotic resistance enzymes, such as NDM-1 and CTX-M-15, which, respectively, confer resistance to most β-lactams (except aztreonam) and cephalosporins (except cephamycins), were detected in the environment, as well as in tap water (10–12). The implications of this escalating preponderance of resistance genes in the environment, and subsequently in human pathogens, cannot be gainsaid, but briefly it portends the end of antibiotics as useful therapeutic agents in combatting bacterial infections (7, 13).

The emergence of antibiotics quickly and significantly reduced the prevalence and mortality of several hitherto fatal infections, such as sepsis, meningitis, tuberculosis, gangrenes, dysentery, food-borne diarrhea, salmonellosis, and pneumonia (1). Increased life-expectancy, better quality of life, and increased wealth were the palpable results of antibiotics use; however, antibiotic resistance remains a threat to these gains. Expansions in resistance among bacteria have been blamed on increased unrestricted antibiotic use in clinical and veterinary medicines (7, 13). Currently, the carbapenems, colistin, and tigecycline, alone or in combinations, are used as reserve antibiotics to treat fatal bacterial infections (14). Unfortunately, reports of resistance to these last-resort antibiotics have been increasing with concerning frequency (15).

Detection of resistance to these last-resort antibiotics in South Africa are increasingly, but worryingly, being reported. To draw attention to this menace, this article provides a comprehensive review of the current burden of resistance to reserve antibiotics, as reported in published literature, as well as discusses their public health implications. This review thus aims to create an awareness of the precarious state of South Africa’s public health as a means of stimulating government action toward implementing policies that will address the situation.

## Last-Resort Antibiotics: From Carbapenems to Colistin and Tigecycline

Until recently, carbapenems, such as meropenem, imipenem, ertapenem, and doripenem, were the last-resort antibiotics used for managing multidrug-resistant bacterial infections (14, 16). However, their use selected for resistant strains, necessitating a change to colistin and tigecycline as last-resort antibiotics (14). It has been established that bacteria develops resistance to carbapenems through the expression/production of enzymes called carbapenemases, and/or the reduction in outer membrane permeability resulting from porin mutations (17). Carbapenemases remain the most clinically important mechanism of carbapenem resistance (18) and are categorized into three classes, namely class A, B, and D; class A includes the clinically important KPC and GES enzymes, while VIM, IMP, and NDM are the most described types under class B. OXA-48-like enzymes remain the commonly reported types under class D (17, 19). Resistance to colistin and tigecycline are, respectively, due to lipid A mutations and efflux hyper expression (14). Recently, Liu et al. (7) found a new plasmid-borne colistin resistance *mcr-1* gene that is quickly spreading worldwide (20). The increasing but worrying emergence of these carbapenemases and carbapenem-, colistin- (particularly *mcr-1*), and tigecycline-resistant Gram-negative bacteria in South Africa are presented herein.

### Resistance to Carbapenems in South Africa

**Figure 1:**
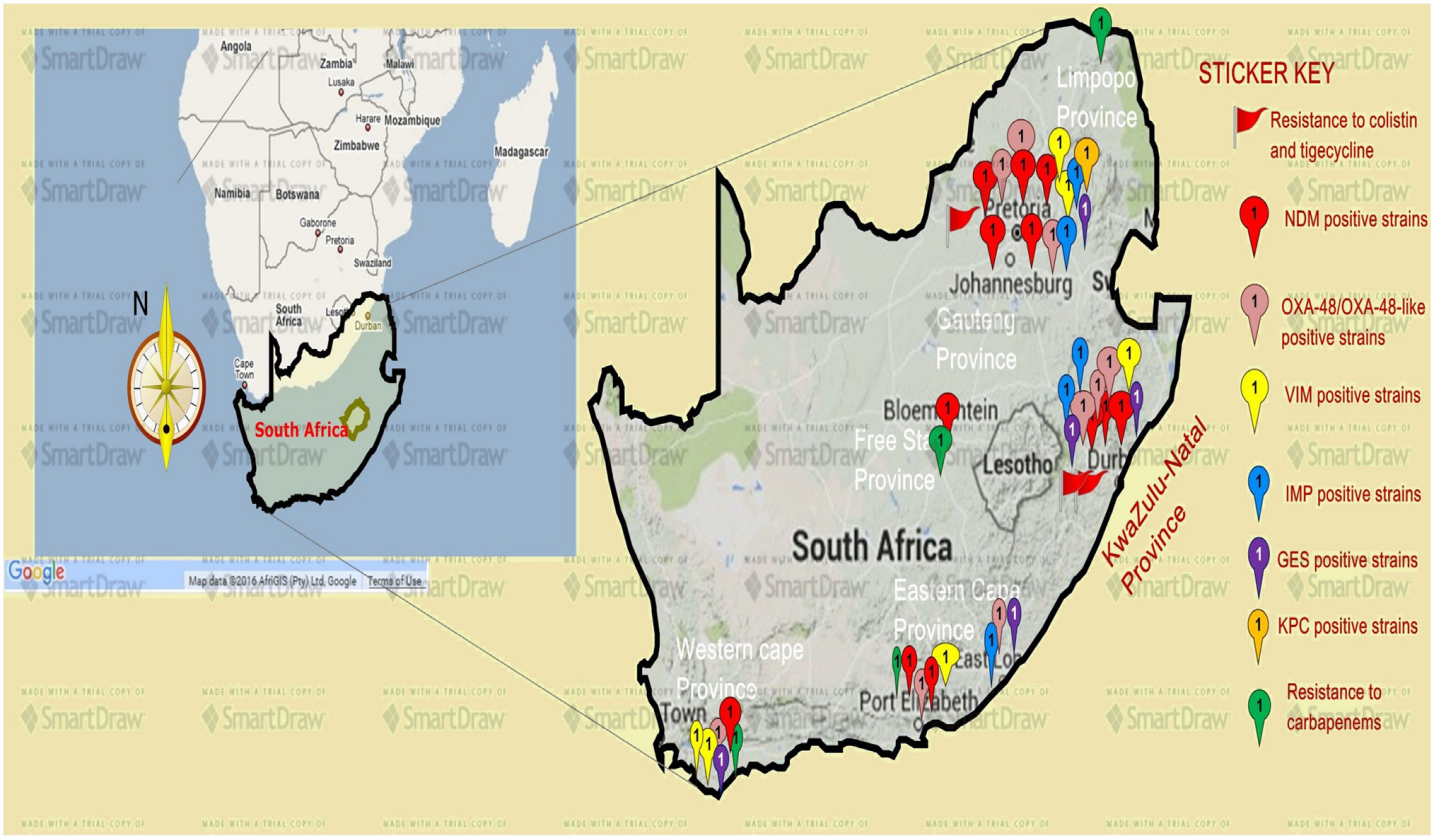
**A map showing the distribution, resistance mechanisms, and density of carbapenem, colistin, and tigecycline resistance described so far in South Africa**. Resistance to carbapenems is highest in the Gauteng and KwaZulu-Natal provinces, followed by the Eastern and Western Capes, respectively. Resistance to colistin (mediated by the mcr-1 gene in some cases) and tigecycline are increasingly also being reported in Gauteng and KwaZulu-Natal provinces. NDM-1 (red ribbons) and OXA-48 (pink ribbons) variants are the commonly reported carbapenem resistance mechanism described in South Africa, particularly in Enterobacteriaceae such as *K. pneumoniae, Enterobacter cloacae, Serratia marcescens*, and *Acinetobacter baumannii*. Relatively minor resistance cases were reported from the Free state and Limpopo provinces, while VIM, IMP, GES, and other carbapenemases are less described in South Africa. NB: a free version of smartdraw software was used to design this map, hence the watermark “smartdraw” in the background.

Analysis of carbapenem resistance-reporting publications showed that an estimated 2315 carbapenem-resistant cases/infections occurred between January 2000 and May 20, 2016 (Tables 1 and 2 and Table S1 in Supplementary Material). The majority of these estimated cases (*n* = 1220) were from the Gauteng province, followed by a substantial number from KwaZulu-Natal (*n* = 515) province (Figure 1). *Klebsiella pneumoniae* (*n* = 1138), *Acinetobacter baumannii* (*n* = 332), *Enterobacter cloacae* (*n* = 201), and *Serratia marcescens* (*n* = 108) were the most common carbapenem-resistant isolates. The most described carbapenemases were NDMs (*n* = 860) and OXA-48 (*n* = 584), which were increasingly detected after 2012, subsequent to its detection in 2011 (Tables 1 and 2 and Table S1 in Supplementary Material; Figure 1). The National Institute of Communicable Diseases (NICD) currently only reports carbapenem-resistant Enterobacteriaceae (CRE) from the public and private health sectors, and not carbapenem-resistant, non-fermenting Gram-negative bacteria or colistin and tigecycline resistance; however, it is on alert to report on the *mcr-1* gene (as at the time of writing this paper). As a reference laboratory, the NICD receives and confirms CRE from selected hospitals from South Africa and publishes them in monthly communiques.

**Table 1:** **Timeline of published carbapenem-resistant Enterobacteriaceae (CREs) and carbapenemases detected in Gram-negative bacteria in South Africa**.

Year	Province (city) (*n*)	Species (*n*)	Specimen type (*n*)	Number of patients (*n*)	Carbapenem resistance mechanism (*n*)	Reference
2000	NS^a^	*Pseudomonas aeruginosa GW-1* (1)	Blood (1)	1	GES-2 (1)	Poirel et al. (21)
2004–2005	Limpopo	*Aeromonas hydrophila* (18)	Stools (309)	309	NC^b^	Obi et al. (22)
2006	Western Cape (Cape Town)	*Klebsiella pneumoniae* (1)	Tracheal aspirate (1)	1	ESBL^c^ + porin deficiency	Elliott et al. (23)
		*K. pneumoniae* (2)	Stool (2)	2	CTX-M-15 + porin deficiency	Segal and Elisha (24)
	NS	*Acinetobacter baumannii* (1)	Urine (1)	1	OXA-23 (1)	Mugnier et al. (25)
2006–2009	Eastern Cape (119)	*Salmonella typhi* (28)	Blood (96)	119	NC	Bisi-Johnson and Obi (26)
2007–2008	NS	*A. baumannii* (1)	NS	1	OXA-51	Zander et al. (27)
2008	Gauteng (Pretoria)	*A. baumannii* (97)	NS	NS	OXA-51 (81), OXA-23 (58), OXA-58 (3), VIM (1)	Kock et al. (28)
2010	Gauteng (Pretoria)	*A. baumannii* (232)	ETA^d^ (149), blood (20), urine (15), CVP^e^ tips (11), wound swabs/tissues/effusions (37)	232	Uncharacterized carbapenemases (217)	Ahmed et al. (29)
2010–2011	Western Cape (Cape Town)	*P. aeruginosa* (15)	Blood (10), stool (2), bile (1), urine (1), catheter (1)	15	VIM-2 (11), GES-2 (1)	Jacobson et al. (30)
2010–2012	Gauteng (75), Western Cape (25), KZN^f^ (15), Free State (7), Limpopo (2)	*K. pneumoniae* (124)	Blood (124)	124	Non-carbapenemase-producing CREs	Perovic et al. (31)
2011	Gauteng	*K. pneumoniae* KPSA01 ST 569 (1)	NS	1	VIM-1 (1)	Peirano et al. (32)
	Gauteng (Johannesburg)	*E. cloacae* (1)	Sputum (1)	1	NDM-1 (1)	Lowman et al. (33)
	Gauteng (Johannesburg, Pretoria)	*K. pneumoniae* (4), *E. cloacae* (1)	Urine (1), tracheal aspirate (1), Blood and tracheal aspirate and catheter tip (3)	2	KPC-2 (4), NDM-1 (1)	Brink et al. (34)
	Western Cape (Cape Town, 4), Eastern Cape (Port Elizabeth, 3), Gauteng (Johannesburg, 2)	*K. pneumoniae* (≥4)	Tissue (2), tracheal aspirate (2), urine (5)	≥4	OXA-48 (2), OXA-181 (2)	Brink et al. (35)
2011–2012	Gauteng (Johannesburg, 105)	*K. pneumoniae, E. cloacae, K. oxytoca, S. marcescens* and *Citrobacter amalonaticus*	Sputum, blood, urine, pus, broncho alveolar lavage, pleural fluid	105	NDM-1 (≥38)	de Jager et al. (36)
2012	KZN (Durban)	*E. cloacae* (1)	Urine (1)	1	NDM-1 (1)	Govind et al. (37, 38)
	KZN (Durban, 4)	*E. cloacae* (2), *C. freundii* (1), *S. marcescens* (1)	Urine (1), sputum (2), tracheal aspirate (1)	4	NDM-1 (4)	Rubin et al. (39)
	Western Cape (Cape Town)	*K. pneumoniae* ST14 (7)	Blood (1), stool (5), pus swab (1), tracheal aspirate (1)	8	OXA-181 (7)	Jacobson et al. (40)
12/2012–10/2013	Gauteng (Johannesburg, 37)	*K. pneumoniae* (17), *E. cloacae* (13), *E. coli* (3), *K. oxytoca* (2), *Klebsiella* spp. (2)	Urine (6), catheter tip (3), blood (2), sputum (1)	37	IMP (5), NDM (4), OXA-48-like (2), VIM (1)	Chibabhai and Perovic (41)
2012–2013	KZN (Durban, 14)	*P. aeruginosa* (8)	Sputum (14)	14	NDM-1 (12)	Mhlongo et al. (42)
2012–2013	KZN (Durban, 48)	*K. pneumoniae* (21), *S. marcescens* (12), *E. cloacae* (11), *C. freundii* (2), *E. coli* (1), *K. oxytoca* (1)	Urine (20), catheter tip (2), blood (4), CVP (4), Pus swab (2), ETA (1), tracheal fluid (3), abdominal fluid/swab (2), art line (1)	46	NDM-1/-5 (33), GES-5 (8), OXA-232 (1)	Osei Sekyere et al. (14, 15)
2013	KZN (Durban)	*E. cloacae* (16), *K. pneumoniae* (9)	Rectal swabs (30)	30	OXA-48 (1)	Govender (43)
	NS	*Acinetobacter nosocomialis* (1)	NS	1	OXA-23	Zander et al. (44)
	KZN (Durban)	*K. pneumoniae* (17), *E. cloacae* (11), *Serratia* spp. (8), *E. coli* (3), *Citrobacter* spp. (2)	Urine (15), respiratory tract samples (9), line tips (6), pus swabs (5), tissue (3), blood (1)	38	NDM-1 (26), GES (6), VIM (1), OXA-48 + NDM-1 (1)	Govind et al. (37, 38)
	KZN (Durban)	*K. pneumoniae* (1)	Endotracheal aspirate (1)	1	NDM-1 (1)	Mahabeer et al. (45)
2015	KZN (Durban)	*K. pneumoniae* (1)	Endotracheal aspirate (1)	1	NDM-1 (1)	Singh et al. (46)

*^a^Not stated in manuscript*.

*^b^Not characterized or determined*.

*^c^Extended spectrum β-lactamase*.

*^d^Endotracheal aspirate*.

*^e^Central venous puncture*.

*^f^KwaZulu-Natal*.

**Table 2:** **Prevalence of carbapenemases and CRE per province as detected by NICD between 2011 and 2015**.

Year	Province	CPE positive specie(s) (*n*)	Carbapenemases/carba penemase negative CREs^a^	Reference(s) (monthly NICD communiqués) National Institute for Communicable Diseases (47)
11/2011	NS	NS (44)	NDM-1 (9)	September 2012 vol. 11 (9)
2011–2013	NS	NS (191)	NDM-1 (37), OXA-48 (8), IMP (6), OXA-48-like (6), KPC (5)	March 2013 vol. 12 (3)
01/2013–04/2014	Easter n Cape (25)	*E. cloacae* (25)	IMP (15), OXA-48 (1), VIM (1)	October 2015 vol. 14 (10)
05–07/2013	Gauteng (10), KZN (4), others (56)	NS	NDM-1 (19)	August 2013 vol. 12 (8)
08–09/2013	Gauteng (42), Western Cape (9), Eastern Cape (4), KZN (3)	*K. pneumoniae* (57), *E. cloacae* (22), *E. coli* (2), *C. freundii* (2), *Morganella morgannii* (1)	NDM-1 (27), OXA-48 (9), OXA-48-like (9), IMP (2), GES (1), VIM (1)	October 2013 vol. 12 (10)
10/2013	Gauteng, KZN, W. Cape	*K. pneumoniae* (14), *E. cloacae* (8), *S. marcescens* (4), *K. oxytoca* (2), *E. coli* (1)	NDM-1 (15), IMP (7), OXA-48-like (6), VIM (1)	November 2013 vol. 12 (11)
11/2013	Gauteng (37), KZN (11), E. Cape (2), W. Cape (1)	*K. pneumoniae* (32), *E. cloacae* (8), *P. rettgeri* (4), *S. marcescens* (2), *Pantoea* (2), *E. aerogenes* (1), *K. oxytoca* (1), *Citrobacter sedlakki* (1)	NDM (16), OXA-48 (14), VIM (12), IMP (6), GES (3)	December 2013 vol. 12 (12)
12/2013	Gauteng (12), KZN (4)	*K. pneumoniae* (10), *E. cloacae* (2), *C. freundii* (2), *E. coli* (1), *S. marcescens* (1)	NDM (9), VIM (3), IMP (2), OXA-48 (2)	January 2014 vol. 13 (1)
01/2014	Gauteng (27)	*K. pneumoniae* (20), *E. cloacae* (2), *E. coli* (2), *C. freundii* (1), *S. marcescens* (1), *P. rettgeri* (1)	NDM (17), VIM (6), OXA-48 (3), GES (1)	February 2014 vol. 13 (2)
02/2014	Gauteng (32), E. Cape (7), W. Cape (4), KZN (3)	*K. pneumoniae* (40), *E. cloacae* (3), *K. oxytoca* (2), *E. coli* (1)	NDM (18), VIM (14), OXA-48 (12), IMP (1), GES (1)	March 2014 vol. 13 (3)
03/2014	Gauteng (30), KZN (7), W. Cape (3), E. Cape (2)	*K. pneumoniae* (32), *E. cloacae* (5), *C. freundii* (2), *S. marcescens* (2), *C. brakii* (1)	NDM (23), OXA-48 (10), VIM (6), GES (2), KPC (1)	April 2014 vol. 13 (4)
04/2014	Gauteng (11), KZN (6), E. Cape (5), W. Cape (1)	*K. pneumoniae* (12), *S. marcescens* (4), *E. cloacae* (3), *C. freundii* (2), *K. oxytoca* (1), *E. aerogenes* (1)	OXA-48 (12), NDM (10), VIM (1)	May 2014 vol. 13 (5)
05/2014	Gauteng (11), KZN (7), W. Cape (3), Free State (1)	*K. pneumoniae* (12), *C. freundii* (4), *S. marcescens* (3), *E. cloacae* (2), *K. oxytoca* (1)	NDM (10), OXA-48 (7), VIM (5)	June 2014 vol. 13 (6)
06/2014	Gauteng (18), KZN (6), E. Cape (1)	*K. pneumoniae* (17), *P. rettgeri* (4), *C. freundii* (1), *E. asburiae* (1), *E. cloacae* (1), *E. coli* (1)	NDM-1 (19), OXA-48 (4), VIM (2)	July 2014 vol. 13 (7)
07/2014	KZN (8), Gauteng (5), E. Cape (1)	*K. pneumoniae* (10), *C. freundii* (3), *E. cloacae* (1)	NDM (8), OXA-48 (3), KPC (1), VIM (1), GES (1)	August 2014 vol. 13 (8)
09/2014^b^	Gauteng (3), KZN (3), E. Cape (3), W. Cape (3)	*K. pneumoniae* (10), *S. marcescens* (2)	NDM (6), OXA-48 (6)	October 2014 vol. 13 (10)
10/2014	Gauteng (17), KZN (16)	*K. pneumoniae* (26), *S. marcescens* (4), *E. cloacae* (2), *Raoultella* spp. (1)	NDM (10), OXA-48 (9), VIM (9), GES (5)	November 2014 vol. 13 (11)
11/2014	KZN (7), Gauteng (6), E. Cape (2), W. Cape (1)	*K. pneumoniae* (10), *S. marcescens* (3), *E. cloacae* (2), *P. rettgeri* (1)	OXA-48 (8), NDM (6), GES (2)	December 2014 vol. 13 (12)
12/2014	Gauteng (29), KZN (13), E. Cape (2), W. Cape (2)	*K. pneumoniae* (24), *S. marcescens* (8), *E. cloacae* (4), Citrobacter complex (4), *P. rettgeri* (2), *Enterobacter gergoviae* (1), *E. asburiae* (1), *E. coli* (1), *M. morgannii* (1)	OXA-48 (19), NDM (18), VIM (5), KPC (3), IMP (1)	January 2015 vol. 14 (1)
01/2015	Gauteng (13), KZN (7), W. Cape (5), E. Cape (1)	*K. pneumoniae* (14), *S. marcescens* (3), *P. rettgeri* (3), *E. cloacae* (2), *E. coli* (2), *K. oxytoca* (1), *Raoultella ornithinolytica* (1)	NDM (16), OXA-48 (8), VIM (1)	February 2015 vol. 14 (2)
02/2015	Gauteng (23), KZN (7), W. Cape (2), E. Cape (1)	*K. pneumoniae* (19), *P. rettgeri* (6), *S. marcescens* (3), *E. coli* (2), *E. cloacae* (1), *K. oxytoca* (1), *Providentia penneri* (1)	NDM (20), OXA-48 (12), VIM (1)	March 2015 vol. 14 (3)
03/2015	Gauteng (34), KZN (18), E. Cape (5)	*K. pneumoniae* (30), *S. marcescens* (7), *E. cloacae* (7), *E. coli* (6), *C. freundii* (2), *K. oxytoca* (2), *P. rettgeri* (1), *E. asburiae* (1)	NDM (36), VIM (12), OXA-48 (9)	April 2015 vol. 14 (4)
04/2015	Gauteng (19), KZN (19), E. Cape (2)	*K. pneumoniae* (26), *E. coli* (5), *S. marcescens* (3), *P. rettgeri* (3), *C. freundii* (2), *E. cloacae* (1)	NDM (27), OXA-48 (13)	May 2015 vol. 14 (5)
05/2015	Gauteng (42), KZN (27), E. Cape (8)	*K. pneumoniae* (48), *E. coli* (9), *S. marcescens* (5), *E. cloacae* (5), *P. rettgeri* (2), *K. oxytoca* (2), *C. freundii* (1)	NDM (48), OXA-48 (23), VIM (4)	June 2015 vol. 14 (6)
06/2015	Gauteng (30), KZN (29), unstated (16), E. Cape (1)	*K. pneumoniae* (52), *S. marcescens* (3), *C. freundii* (2), *E. coli* (1), *E. cloacae* (1), *M. morgannii* (1)	NDM (45), carbapenemase-negative CREs (16), OXA-48 (9), VIM (6)	July 2015 vol. 14 (7)
07/2015	KZN (32), unstated (22), Gauteng (19), E. Cape (5), unknown (2)	*K. pneumoniae* (45), *S. marcescens* (6), *K. oxytoca* (6), *E. coli* (3), *E. cloacae* (1), *C. freundii* (1)	NDM (46), carbapenemase-negative CREs (22), VIM (9), OXA-48 (7)	August 2015 vol. 14 (8)
08/2015	KZN (17), Gauteng (11), E. Cape (8), unstated (8), Free State (1)	*K. pneumoniae* (30), *S. marcescens* (3), *E. coli* (2), *E. cloacae* (1), *C. freundii* (1)	NDM (20), OXA-48 (12), carbapenemase-negative CREs (8), VIM (5)	September 2015 vol. 14 (9)
09/2015	KZN (14), Free State (11), E. Cape (11), Gauteng (8), unstated (3), W. Cape (2)	*K. pneumoniae* (35), *S. marcescens* (5), *E. coli* (2), *E. cloacae* (2), *P. rettgeri* (1)	NDM (33), OXA-48 (12), carbapenemase-negative CREs (11)	October 2015 vol. 14 (10)
10/2015	Gauteng (39), KZN (31), E. Cape (7), Free State (2)	*K. pneumoniae* (49), *K. oxytoca* (3), *S. marcescens* (2), *E. cloacae* (2), *E. coli* (1), *C. freundii* (1)	NDM (34), OXA-48 (21), VIM (3), carbapenemase-negative CREs (19)	November 2015 vol. 14 (11)
11/2015	Gauteng (55), unstated (20), KZN (7), E. Cape (4), W. Cape (4), Free State (4)	*K. pneumoniae* (43), *E. coli* (13), *S. marcescens* (7), *E. cloacae* (4), other Enterobacteriaceae (4), *C. freundii* (3), *K. oxytoca* (2), *P. rettgeri* (2)	NDM (38), OXA-48 (35), VIM (5), carbapenemase-negative CREs (16)	December 2015 vol. 14 (12)
12/2015	Gauteng (71), KZN (31), E. Cape (16), W. Cape (7), Free State (5)	*K. pneumoniae* (84), *E. cloacae* (9), *S. marcescens* (5), *P. rettgeri* (3), *C. freundii* (2), *E. coli* (1), *E. kobei* (1), *M. morganii* (1)	NDM (53), OXA-48 (47), VIM (4), GES (1), KPC (1), carbapenemase-negative CREs (14)	January 2016 vol. 15 (1)
01/2016	Gauteng (34), KZN (18), E. Cape (12), W. Cape (11), Free state (1)	*K. pneumoniae* (43), *E. cloacae* (7), *S. marcescens* (5), *E. coli* (3), *P. rettgeri* (3)	NDM (29), OXA-48 (32), carbapenemase-negative CREs (15)	February 2016 vol. 15 (2)
02/2016	W. Cape (78), Free state (34), E. Cape (21), Gauteng (12), KZN (11)	*K. pneumoniae* (59), *E. cloacae* (8), *S. marcescens* (2), *C. freundii* (2), *P. mirabilis* (1), *E. coli* (1), *E. kobei* (1), *M. morganii* (1), *K. oxytoca* (1), *E. aerogenes* (1), *Citrobacter amalonaticus* (1)	NDM (25), OXA-48 (36), carbapenemase-negative CREs (17)	March 2016 vol. 15 (3)

*^a^Carbapenem-resistant Enterobacteriaceae*.

*^b^Several isolates were uncharacterized due to technical difficulties (NICD)*.

These observations suggest the substantial dissemination of carbapenem resistance, mediated mostly by NDM-1 or OXA-48 carbapenemases in *K. pneumoniae, A. baumannii, E. cloacae, S. marcescens*, and other Gram-negative bacteria. After 2000 A.D., the number of carbapenem resistance reports increased in South Africa, specifically in Gauteng and KwaZulu-Natal provinces (Tables 1 and 2). Reports of NDM and OXA-48-like carbapenemases increased after 2011 in Gauteng, KwaZulu-Natal, Eastern, and Western Capes provinces (Tables 1 and 2; Figure 1). Most of these carbapenem resistance cases had no travel history outside South Africa, suggesting that these strains were selected from increased carbapenem use within the country (48). Published articles and the NICD communiqués tend to agree in a large measure with regards to the detection patterns and distribution of carbapenemases and CREs among the provinces (Table S1 in Supplementary Material). Carbapenem resistance has been described in at least 10 Gram-negative bacterial hosts/species, which have been isolated from various clinical specimens (Tables 1 and 2); but with the exception of *mcr-1* that was found in *Escherichia coli* in poultry (49), no reports of carbapenem- or tigecycline-resistant isolates were found from animals.

Reports of carbapenem-resistant Enterobacteriaceae from animals or the environment were not seen, and the NICD does not survey or collect CRE from livestock or environmental sources. Given the detection of mcr-1 among poultry and humans (50) in two provinces in South Africa, the need for a “One Health” approach to antimicrobial resistance surveillance and genomic epidemiology is dire (8). It is particularly concerning that these data are not representative of the whole burden of carbapenem resistance in South Africa. Subsequently, the burden of carbapenem resistance could therefore be more than reported, should all hospitals and laboratories, veterinarians, and environmentalists/ecologists report their carbapenem-resistant cases. In addition, the distribution, resistance mechanisms, and bacterial hosts could change with a holistic “One Health” approach.

Given the high attributable mortalities resulting from carbapenem-resistant, and especially NDM-positive bacterial infections (51, 52), their public health impacts cannot be over emphasized, particularly as studies indicate that many CRE-infected South African patients demised (35, 40, 53). Given the persistent and undetected nature of these carbapenem-resistant strains, specifically in many South African hospitals, the possibility of outbreaks, interhospital and intrahospital circulation (30, 40), and their transmission *via* hospital staff and patients to homes and communities is very high. Due to the association of CREs with intensive care units and invasive medical procedures, all patients who patronize these services are highly at risk (34, 54). With the detection of carbapenem resistance among *Salmonella typhi* in a hospital in Port Elizabeth, CREs can easily be transferred *via* contaminated food and water, a route that will expose many to these germs (26). This is further evidence of the need for a “One Health” approach to the antibiotic resistance problem in South Africa as resistant bacteria are not only reserved in clinical settings.

The escalating prevalence of these resistant strains is not only significant for South Africa but also to Africa and Europe as a whole, as published evidence shows that many patients from across Africa and Europe are transferred to South Africa for better and cheaper medical attention. For instance, it is established that many Europeans troop to South Africa as a destination for cosmetic surgeries, such as hip replacements, rhinoplasty, breast augmentation, liposuction, facelifts, and tummy tucks (55); the possibility of South Africa thus spreading these resistant strains to other African and European nations cannot be ruled out as medical tourism in South Africa increased from 3.9 to 5.0% of all entries between 2006 and 2010 (55). This should alert the government, the health-care industry, and health workers to the economic costs associated with the increasing prevalence of CREs as it could deter patients from across Africa and Europe from seeking health care in the country.

The main challenge regarding the combatting of these resistant strains lies in the limited antibiotics available for their treatment. In KwaZulu-Natal, CREs have been isolated from neonates, including a 24-h neonate (45, 46) for whom antibiotic therapy are further limited due to their age. Clinicians are then forced to resort to more expensive but toxic combination therapies that include the nephrotoxic and neurotoxic colistin and/or tigecycline (56, 57), and to extend the patient’s stay in the hospital. The cost arising from the extended hospital stay to the patient, their family, and the government, as shown by the WHO in the US, EU, and Thailand, are considerable (1). Moreover, hospitals with outbreaks of CREs need to spend considerable resources sterilizing all wards and instituting stricter infection management controls, with some being forced to close down until the outbreaks are traced and eradicated (40). It is therefore in the interest of hospitals to institute periodic surveillance and effective infection control measures to preempt full-scale outbreaks, as well as screen all incoming patients to quickly isolate carriers.

### Increasing Resistance to Tigecycline and Colistin

The exponential consumption of carbapenems in South Africa between January 2009 and June 2011, due to rising extended-spectrum β-lactamases among invasive Enterobacteriaceae, was early signaled as a potential factor in selecting CREs (14, 49, 54). It has been shown that colistin use in South Africa is increasing rapidly, and the failure of the carbapenems has been fingered as a cause (48, 49). Therefore, the increasing use of colistin and tigecycline due to an ever-expanding prevalence of CREs will result in colistin resistance (14, 58) as has been already observed among 45 CREs, 40 of which were resistant to both colistin and tigecycline (15), and also reported by Brink et al. (35) in *K. pneumoniae* (35). With the emergence of colistin resistance among CREs, the pre-antibiotic age, due to pandrug resistance, is not far off. While reports of colistin- and tigecycline-resistant CREs in South Africa are relatively few, rather than suggesting their low prevalence, it indicates the absence of surveillance to detect them.

For instance, surveillance for *mcr-1* in poultry and patients led to the first detection of this gene in multiclonal *E. coli* strains in Gauteng and Western Cape provinces (49). This is an unfortunate finding, from a public health perspective, as consumption of the infected poultry can quickly spread *mcr-1* among consumers and further compromise colistin therapy in CRE-infected patients in South Africa. The selection of *mcr-1* among the poultry was suggested as due to the high use of colistin among South African poultry as growth promoters, prophylactic, and therapeutic agents, underscoring the need for stricter regulations on antibiotic use in the veterinary sector. Being found in *E. coli*, it is not surprising that the affected patients included outpatients; this is due to the common presence of *E. coli* in both community and hospital settings. This further portrays an ominous future as *E. coli* and the plasmid-borne *mcr-1* gene can easily find their way into environmental sources through humans and animals waste. Moreover, other bacterial species can easily obtain this gene from *E. coli* through the *mcr-1* plasmid, leading to further spread (7). The higher prevalence of colistin and tigecycline resistance recorded in Durban (15) suggests that it remains largely undetected and underscores the need for further surveillance studies in hospitals, farms, and environments. Without colistin and tigecycline (there are no other reserve antibiotics), the prognosis of bacterial infections in South Africa would be bleak.

Colistin and tigecycline resistance cases were higher in KwaZulu-Natal province (Figure 1) than in Gauteng province and were common among *K. pneumoniae, A. baumannii, S. marcescens*, and *E. cloacae* (Table 3), albeit *mcr-1* has been detected in Gauteng (Johannesburg) and Western Cape (Cape Town) provinces so far (49). In the abovementioned study, 44 CREs were resistant to tigecycline; 41 of which were resistant to colistin, with 40 being resistant to both colistin and tigecycline (15) (Table 3). The first *mcr-1* gene to be detected in South Africa was hosted in polyclonal *E. coli* strains isolated from poultry (*n* = 19) and from outpatients and inpatients in Gauteng and Western Cape provinces (49).

**Table 3:** **Reported/published cases of resistance to colistin and tigecycline in South Africa**.

Year	Province	Species (*n*)	Number of patients	Reference
**Tigecycline resistance**				
2010	Gauteng (Pretoria)	*A. baumannii* (17)	17	Ahmed et al. (29)
2012–2013	KZN (Durban)	*K. pneumoniae* (19), *S. marcescens* (12), *E. cloacae* (11), *C. freundii* (1), *E. coli* (1)	44^a^	Osei Sekyere et al. (14, 15)
2013	KZN (Durban)	Enterobacteriaceae (12)	12	Govind et al. (37, 38)
**Colistin resistance**				
2011	Gauteng (Johannesburg)	*K. pneumoniae* (11)	1	Brink et al. (35)
2012–2013	KZN (Durban)	*K. pneumoniae* (17), *S. marcescens* (12), *E. cloacae* (10), *K. oxytoca* (1), *E. coli* (1)	41^a^	Osei Sekyere et al. (14, 15)
2016	Gauteng (Johannesburg and Pretoria)	*E. coli* (28)	9 (19 were from poultry)	Coetzee et al. (49)
	Western Cape (Cape Town)			

*^a^Forty of these were also resistant to colistin and carbapenems*.

The geographical location of the carbapenem-, colistin-, and tigecycline-resistant strains may not necessarily suggest their higher prevalence in Gauteng, KwaZulu-Natal, and the Eastern, and Western Cape provinces, but rather a higher surveillance effort in these areas. However, the higher risk posed to the citizens of these provinces cannot be downplayed, as these pandrug-resistant strains could be easily spread into the environment and community (12, 13). The broad species range (Tables 1–3 and Table S1 in Supplementary Material) with which these carbapenem resistances are associated are also worrying, as these bacteria are commonly implicated in many human illnesses. Thus, the absence of reserve antibiotics in treating common infections from these resistant human pathogens will be precarious to public health.

### Necessary Remedial and Policy Interventions

The escalating reports of NDMs, estimated at 34% in 2014 to 59% in 2015 (59), and other carbapenemases in addition to the inadequate national data, belie the porous infection control measures and surveillance programs in South Africa. This must change if the resistance menace is to be contained. A stricter enforcement of antibiotic use regulations among prescribers, dispensers, and livestock farmers is warranted to reduce the amount consumed in South Africa (48). It is incumbent on government and health regulators to enforce antibiotic stewardship programs as well as institute weekly or fortnight surveillance in all hospitals in addition to well-established livestock and environmental surveillance. Anal swab screening of incoming and hospitalized patients, as well as screening of hospital staff, wards, and all invasive medical instruments should be enforced in all hospitals. Food animals must be periodically sampled and tested for resistant bacteria. Contact precautions and patient isolation protocols, as advised by the Centers for Disease Control and Prevention (CDC), should be taught to health workers and strictly implemented for infected patients (60). In addition, patients returning from high-risk countries (with higher prevalence of carbapenem, colistin, or tigecycline resistance) should be screened before admission (60).

Currently, the NICD monitors and receives CRE from selected hospitals in South Africa, which it uses for its monthly communiques (Table 2). This is a good step toward describing the trends in CRE prevalence in South Africa, albeit it falls short of establishing their molecular epidemiology that is pivotal for any meaningful intervention. Moreover, efforts should be made to include all hospitals, in both private and public sectors, livestock farms, and environments, to obtain a true picture of the situation in the country. To further enhance epidemiological analysis, isolates included in published manuscripts should be removed from the NICD communiques or flagged therein to avoid their being double counted. In addition, the NICD should collaborate with universities and hospitals to establish “Centers for Genomic Epidemiology of Resistant Infections” to train and equip students, clinicians (physicians and veterinarians), and environmentalists/ecologists to undertake periodic antibiotic resistance surveillance and genomic epidemiology studies in hospitals, the environment, and livestock farms under a “One Health” (8) approach using next-generation sequencing (NGS) technology. This will help trace the routes and describe the evolutionary biology of resistant infections over time, which will provide adequate data to inform intervention policies.

There should be periodic in-service training for all hospital staff to increase awareness about carbapenem, colistin, and tigecycline resistance and the need to reduce their use. Such training must also involve the imparting of CRE diagnosis or detection skills and education in the available detection tools and methods as well as results interpretation. Hence, skills in undertaking and interpreting antimicrobial sensitivity tests using “interpretative reading” (61), the Carba NP test, multiplex real-time PCR, and/or whole genome sequencing will be useful.

Furthermore, prescribers should be encouraged to adopt evidence-based therapies to reduce antibiotic prescriptions, specifically for non-bacterial-based infections. Dispensers and community pharmacies must only serve antibiotics under prescription, with regulations to punish offending practitioners (1, 2). Government, the media, and schools must complement the efforts of the WHO by broadcasting antibiotic awareness days and weeks throughout South Africa, to alert the inhabitants of the need to finish their antibiotic courses, avoid buying antibiotics without prescriptions, avoid sharing their unfinished antibiotics with family and friends, and/or avoid advising friends or family to buy antibiotics when they develop symptoms similar to that of a known disease treated with a particular antibiotic (1, 2).

## Conclusion

There is an escalating prevalence and possible endemicity of NDM and OXA-48 among Enterobacteriaceae and *A. baumannii* in South Africa, which is largely under-detected. Resistance to colistin, mediated by the plasmid-borne *mcr-1* gene and other chromosomal mutations, and tigecycline is burgeoning among Gram-negative bacteria, leaving clinicians with no reserve antibiotics for treating fatal bacterial infections. Stricter infection control and antibiotic stewardship, “One Health” surveillance and genomic epidemiology studies, education, and awareness creation are warranted.

## Author Contributions

This paper was designed, prepared, and finalized by John Osei Sekyere, Ph.D.

## Conflict of Interest Statement

The author declares that the research was conducted in the absence of any commercial or financial relationships that could be construed as a potential conflict of interest.
