# Supplementary material for: Current State of Resistance to Antibiotics of Last-Resort in South Africa: A Review from a Public Health Perspective
Source: Front Public Health. 2016 Sep 30;4:209. doi: 10.3389/fpubh.2016.00209 (PMC5042966; doi:10.3389/fpubh.2016.00209)
Supplement: Supplementary file 1 [file table_1.docx]

Table S1: Summary of carbapenem resistance mechanisms per province and bacterial species.

| Provinces/species/carbapenemase | Published manuscripts | NICD communiqués | Total |
| --- | --- | --- | --- |
| **Recorded provinces** | | | |
| Gauteng | 514 | 706 | 1220 |
| KwaZulu-Natal | 146 | 369 | 515 |
| Eastern Cape | 122 | 156 | 278 |
| Western Cape | 55 | 138 | 193 |
| Free state | 7 | 59 | 66 |
| Unknown | - | 23 | 23 |
| Limpopo | 20 | - | 20 |
| **Recorded species** | | | |
| *K. pneumoniae* | 235 | 903 | 1138 |
| *A baumannii* | 332 | - | 332 |
| *E. cloacae* | 60 | 141 | 201 |
| *S. marcescens* | 15 | 93 | 108 |
| *E. coli* | 7 | 60 | 67 |
| *C. freundii* | 3 | 34 | 37 |
| *P. rettgeri* | - | 36 | 36 |
| *Salmonella typhi* | 28 | - | 28 |
| *K. oxytoca* | 5 | 21 | 26 |
| *P. aeruginosa* | 24 | - | 24 |
| *Aeromonas hydrophilia* | 18 | - | 18 |
| *Serratia spp.* | 8 | - | 8 |
| *Citrobacter spp./complex* | 2 | 4 | 6 |
| *M. morgannii* | - | 4 | 4 |
| Other Enterobacteriaceae | - | 4 | 4 |
| *E. asburiae* | - | 3 | 3 |
| *Pantoea spp.* | - | 2 | 2 |
| *E. aerogenes* | - | 3 | 3 |
| *Citrobacter sedlakki* | - | 1 | 1 |
| *Raoultella spp.* | - | 1 | 1 |
| *Raoultella ornithinolytica* | - | 1 | 1 |
| *Enterobacter gergoviae* | - | 1 | 1 |
| *Enterobacter kobe* | - | 1 | 1 |
| *Providentia penneri* | - | 1 | 1 |
| *Acinetobacter nosocomialis* | 1 | - | 1 |
| *Citrobacter amalonitus* | 1 | 1 | 2 |
| **Reported carbapenemases** | | | |
| NDM | 122 | 738 | 860 |
| OXA-48 (including OXA-48-like enzymes) | 160 | 424 | 584 |
| VIM | 15 | 116 | 131 |
| IMP | 5 | 40 | 45 |
| GES | 16 | 17 | 33 |
| KPC | 4 | 11 | 15 |

# Appendix

# A. Methods (Experimental procedures)

Pubmed, Google Scholar, Sabinet and the National Institute of Communicable Diseases (NICD)^[[1]](#footnote-1)^ (56) databases were searched with the keywords “carbapenem resistance in South Africa”, “carbapenemases in South Africa”, “colistin resistance in South Africa”, tigecycline resistance in South Africa” and “colistin AND tigecycline resistance in South Africa” for publications between early January 2000 to 20^th^ May, 2016. English abstracts, thesis/dissertations and NICD monthly reports (NICD-NHLS Communicable Disease Communiqué) from September 2012 to March 2016, and published papers, ECCMID and FIDSSA conference presentations and posters within the time frame were added. Review papers, in addition to research publications that did not report carbapenem, colistin or tigecycline resistance/resistance mechanisms in bacteria as well as the number, specimen and geographical source of isolates, were excluded. Abstracts that had been published as main research papers were equally excluded.

The data were categorized into two: 1) Publication findings, including abstracts, thesis, conference proceedings and research articles, and (2) NICD monthly communiqués, which report on carbapenem resistant Enterobacteriaceae (CRE) in South Africa. The NICD communiqués (56) were analyzed in parallel to the publication findings to serve as a positive control, as well as to trace the detection of CREs from an official source. A case was defined as the incidence/detection of a carbapenem, colistin and/or tigecycline resistant bacterial isolate in a patient. Where the number of CREs or Gram-negative bacteria was not provided, but the number of carbapenemase-producing Enterobacteriaceae (CPE) or Gram-negative bacteria was, (Tables 1 and 2), the latter was used in the analysis with a “≥” prefix (Tables 1-2).

# B. Results of literature search: inclusion and exclusion criteria

The literature review yielded 104 potential studies/manuscripts that addressed carbapenem resistance. Seventy-two articles were excluded, as they addressed non-South African isolates and/or provided very confusing information (which made it impossible to identify the South African isolates’ data), repeated data from other publications or addressed treatment guidelines, pharmacokinetics or antibiotic stewardship. Four publications were further excluded from the remaining 31 due to insufficient data on patients, specimen types, species and isolate numbers. Twenty-seven publications, which included one master’s dissertation, six abstracts and 20 full-texts, were finally included in the review (Tables 1 and 3).

Seven publications addressing colistin and tigecycline resistant bacteria in South Africa were obtained from the literature search. Two were excluded due to an unspecific number and/or identity of resistant strains, species and specimen types. Five manuscripts, including two abstracts, were used for the analysis (Table 3). None of the NICD’s 32 CRE communiqués that have been published so far, from September 2012 to March 2016, were excluded (Table 3).

1. The NICD is a division of the National Health Laboratory Service (NHLS) in South Africa [↑](#footnote-ref-1)
